# Proteomic analysis of urinary extracellular vesicles of kidney transplant recipients with BKV viruria and viremia: A pilot study

**DOI:** 10.3389/fmed.2022.1028085

**Published:** 2022-11-17

**Authors:** Maurizio Bruschi, Simona Granata, Giovanni Candiano, Andrea Petretto, Martina Bartolucci, Gian Marco Ghiggeri, Giovanni Stallone, Gianluigi Zaza

**Affiliations:** ^1^Laboratory of Molecular Nephrology, IRCCS Istituto Giannina Gaslini, Genova, Italy; ^2^Nephrology, Dialysis and Transplantation Unit, Department of Medical and Surgical Sciences, University of Foggia, Foggia, Italy; ^3^Renal Unit, Department of Medicine, University Hospital of Verona, Verona, Italy; ^4^Core Facilities—Clinical Proteomics and Metabolomics, IRCCS Istituto Giannina Gaslini, Genoa, Italy; ^5^Division of Nephrology, Dialysis and Transplantation, IRCCS Istituto Giannina Gaslini, Genova, Italy

**Keywords:** BK virus, proteomics, kidney transplant recipients, extracellular vesicles, mass spectrometry

## Abstract

**Introduction:**

To better define the biological machinery associated with BK virus (BKV) infection, in kidney transplantation, we performed a proteomics analysis of urinary extracellular vesicles (EVs).

**Methods:**

Twenty-nine adult kidney transplant recipients (KTRs) with normal allograft function affected by BKV infection (15 with only viremia, 14 with viruria and viremia) and 15 controls (CTR, KTRs without BKV infection) were enrolled and randomly divided in a training cohort (12 BKV and 6 CTR) used for the mass spectrometry analysis of the EVs (microvesicles and exosomes) protein content and a testing cohort (17 BKV and 9 CTR) used for the biological validation of the proteomic results by ELISA. Bioinformatics and functional analysis revealed that several biological processes were enriched in BKV (including immunity, complement activation, renal fibrosis) and were able to discriminate BKV vs. CTR. Kinase was the only gene ontology annotation term including proteins less abundant in BKV (with SLK being the most significantly down-regulated protein). Non-linear support vector machine (SVM) learning and partial least squares discriminant analysis (PLS-DA) identified 36 proteins (including DNASE2, F12, AGT, CTSH, C4A, C7, FABP4, and BPNT1) able to discriminate the two study groups. The proteomic profile of KTRs with BKV viruria alone vs. viremia and viruria was quite similar. Enzyme-linked immunosorbent assay (ELISA) for SLK, BPNT1 and DNASE2, performed on testing cohort, validated proteomics results.

**Discussions:**

Our pilot study demonstrated, for the first time, that BKV infection, also in the viruric state, can have a negative impact on the allograft and it suggested that, whether possible, an early preventive therapeutic strategy should be undertaken also in KTRs with viruria only. Our results, then, revealed new mechanistic insights into BKV infection and they selected potential biomarkers that should be tested in future studies with larger patients’ cohorts.

## Introduction

To prevent allograft rejections with an accelerated graft failure, kidney transplant recipients (KTRs) need to undertake a multi-drugs therapy comprising induction (T cell-depleting or interleukin (IL) 2 receptor-blocking antibodies) and maintenance immunosuppressive agents (calcineurin inhibitors, mammalian target of rapamycin, antimetabolites, corticosteroids) used in various combination regimens. Although indispensable, these drugs may induce severe side effects/toxicities and clinical complications (mainly malignancies and infections).

In the early phase after transplantation (<1 year), kidney transplant patients, particularly in case of over-immunosuppression, may develop BK virus (BKV) infection (1, 2). BK is a virus belonging to the Polyomaviridae family with dsDNA (3) that induces a common viral infection in children without residual complications and remains latent in the renal epithelium of healthy subjects. After kidney transplantation, BKV may reactivate leading to viruria in 30–40%, viremia in 10–20% and BKV-associated nephropathy (BKVAN) in about 5% of patients (4–7). BKVAN is associated with a significantly decrease of the graft survival (8).

BKV viruria is the consequence of lysis of infected cells that induces leakage of virus into the tubular lumen. Denudation of the urogenital basement membrane that occurs with high levels of BKV viruria, causes vascular spread and consequent BKV viremia. Persistent high viremia may result in BKVAN (1). Biological events such as DNA damage, apoptosis and release of immune mediators may contribute to the severe inflammation leading to BKVAN (1, 9, 10).

BK viruria precedes viremia (by about 4 weeks) as well as viremia precedes BKVAN (by about 8 weeks) (2, 4). Therefore, by applying real-time polymerase chain reaction (PCR), BKV replication can be identified before nephropathy development with the chance of reducing/minimizing immunosuppression and preventing a rapid allograft morphological damage and functional impairment.

It has been demonstrated that BKV PCR is a useful non-invasive test to identify BKV viruria (> 1 × 107 copies/mL) in urine and viremia (> 1 × 104 copies/mL) in plasma for concomitant BKVAN with a sensitivity of 100% and a specificity of 78 and 92%, respectively (11). The 2019 American Society of Transplantation Infectious Disease Community of Practice (AST-IDCOP) guidelines recommended this method of screening (12).

However, although the clinical impact of BKV viremia has been well defined, at the moment, the exact biological role of the BKV viruria is still poorly defined.

Therefore, to add new insights into the molecular mechanisms underlying the post-transplant BKV infection (also in those with BKV viruria alone) and to identify systemic factors associated with this disease, we applied an untargeted mass spectrometry to compare the protein content of urinary EVs (microvesicles/exosomes) isolated from KTRs with normal renal function and BKV infection vs. CTR (KTRs without infection).

EVs are a source of biomarkers for several kidney diseases (13, 14) and a novel tool to monitor the allograft function. Changes in their composition may reflect ongoing acute and chronic pathological events (15–17).

## Materials and methods

### Patients

In this research pilot study, after obtaining informed consent, we included 44 stable adult deceased donor KTRs who had undergone renal transplantation within at least 3 months before enrollment.

From the entire cohort, 29 KTRs were affected by BKV infection (15 patients with BK viruria alone and 14 patients with both BK viruria and viremia) and 15 controls without infection (CTR) matched for demographic characteristics (age, gender) (Table 1). All patients and controls had normal renal function without hematuria, proteinuria, leukocyturia or other abnormalities of urinary sediment. For this reason, no allograft biopsies were performed (according to our center clinical protocol).

**TABLE 1:** Demographic and clinical characteristics of the study groups.

	BK viruria only	BK viremia and viruria	Controls	*P*-value
Patients (n)	15	14	15	NS
Age (years)	43.2 ± 9.3	46.4 ± 7.4	47.3 ± 7.1	0.35
Gender (M/F)	9/6	8/6	8/7	0.93
Baseline nephropathy: ESKD NOS, glomerulonephritis, interstitial nephritis, nephroangiosclerosis, obstructive uropathy (n)	7/2/2/2/2	6/2/2/1/3	6/1/3/2/3	0.9
Time since transplantation (months)	9.1 ± 3.3	8.7 ± 4.1	8.9 ± 3.9	0.89
Retransplant (Y/N)	2/13	3/11	2/13	0.46
HLA missmatch (mean ± SD)	3.4 ± 1.2	3.5 ± 1.0	3.6 ± 0.9	0.86
Serum Creatinine (mg/dl)	0.89 ± 0.08	0.91 ± 0.07	0.88 ± 0.09	0.60
24 h urine creatinine (mg/die)	1104.41 ± 233.64	1212.34 ± 124.13	1126.91 ± 114.16	0.21
Daily proteinuria (g/24 h)	0.07 ± 0.03	0.08 ± 0.04	0.05 ± 0.06	0.20
Body mass index (BMI) (kg/m^2^)	22.6 ± 4.1	24.1 ± 4.9	23.7 ± 5.1	0.67
Hypertension (Y/N)	2/13	1/13	13/2	0.36
Tacrolimus trough level at enrollment (ng/ml)	8.91 ± 0.51	9.08 ± 0.37	8.99 ± 0.42	0.58

Values are expressed as mean ± SD. ESKD, end-stage kidney disease; NOS, not otherwise specified. *P*-values determined by ANOVA and chi-square test.

A test is considered positive for BKV if the number of viral copies, assessed by quantitative real-time PCR, are more than 10^7^ copies/mL in urine or 10^4^ copies/mL in blood; a positive screening test was confirmed within 4 weeks in both blood and urine. Blood and urine BKV detection were performed on the same day in our Hospital-University Laboratory.

The initial cohort was randomly divided into a training cohort (comprising 6 KTRs with BK viruria and total absence of viremia, 6 with both BK viremia-viruria and 6 CTR) used for proteomics analysis of urinary extracellular vesicles (EV) and a validation cohort (9 KTRs with BK viruria and total absence of viremia, 8 with both BK viremia-viruria and 9 CTR) used for confirming the proteomic results by ELISA.

For all the KTRs, the maintenance immunosuppressive therapy included: twice-daily tacrolimus b.i.d. (trough levels 6–10 ng/ml) in combination with mycophenolate mofetil 1,000 mg b.i.d. and methylprednisolone 4 mg/day. As induction therapy, patients received 500 mg of methylprednisolone intra-operatively, 250 mg of prednisone daily, with the dose tapered to 25 mg by day 8; 20 mg of a chimeric monoclonal anti-CD25 antibody (basiliximab) intravenously on day 0 and day 4.

Patients with a previous or ongoing acute rejection, delayed graft function or surgical complications, and those with other viral infections (such as cytomegalovirus and Epstein-Barr virus), acute or recurrent urinary tract infections, diabetes (including post-transplant diabetes mellitus), gastrointestinal disorders, or malignancies were not enrolled in the study. In addition, patients treated with antibiotics or non-steroidal anti-inflammatory drugs during the previous month before enrollment were excluded.

The study was carried out according to the Declaration of Helsinki and was approved by the Ethics Committee of Verona and Rovigo provinces, Italy (1745CESC).

### Isolation of urinary microvesicles and exosomes

Microvesicles and exosomes were isolated by sequential centrifugal ultrafiltration of second morning urine as previously reported (18). Urine samples (30 ml) were centrifuged at 7,500 × g for 30 min at 16°C to remove cells, debris and organelles. The supernatant was centrifuged at 22,000 × g for 2 h at 16°C to obtain the microvesicle fraction which was, then, resuspended in phosphate-buffered saline (PBS) and centrifuged again at 22,000 × g for a total of five times. The pellet of the final centrifugation step contained the cleaned microvesicle fraction. The supernatant was centrifuged at 100,000 × g for 2 h at 16°C to pellet the exosomes. The pellet, resuspended in 1 ml 0.25 M sucrose, was loaded on 30% sucrose cushion and centrifuged at 100,000 × g for 2 h at 16°C. To obtain a pure exosomal fraction the pellet was subsequently washed five times in PBS and centrifuged always at 100,000 × g for 10 min at 4°C. Microvesicle and exosomal fractions were stored at –80°C until use.

### Mass spectrometry

Microvesicles and exosomes (1 μg) were lysed, reduced and alkylated in iST-LYSE buffer (PreOmics) following the manufacturer’s instructions and further sonicated using an Ultrasonic Processor UP200St (Hielscher) for 3 cycles (30 s on, 30 s off). Samples were digested overnight at 37°C with Trypsin/LysC and, then, processed following iST protocol (19). EASY spray column (75 μm x 50 cm, 2 μm particle size, Thermo Scientific) was used to elute the digested samples with a non-linear gradient of 7–45% solution B (80% acetonitrile, 5% dimethyl sulfoxide, 0.1% formic acid) in 70 min at a 250 nl/min flow rate. Mass spectrometry (MS) analysis was performed in data dependent acquisition (DDA) mode. MS1 measurements were acquired at a resolving power of 120 K between 375 and 1,500 m/z, and an automatic gain control (AGC) target of 400,000 and maximum injection time of 50 ms. Advanced Peak Determination was enabled for MS1 measurements. MS/MS spectra were acquired at a resolution of 30 K and an AGC target of 50,000, maximum injection time of 54 ms, after higher energy collision dissociation (HCD) at energy of 28%. Data dependent MS/MS analysis was performed with a 2 sec. cycle time, the quadrupole isolation setting was 1.6 m/z isolation and dynamic exclusion was enabled for 30 s.

To analyze the raw data MaxQuant software (version 1.6.17.0) was used setting a false discovery rate (FDR) of 0.01 for the identification of proteins, peptides and peptide-spectrum match (PSM). For peptide identification, a minimum of 6 amino acids was required. To search MS/MS spectra against Uniprot human database (release UP000005640_9606 April 2020) and Uniprot BK virus database (release BK_Plyomavirus_000008166 November 2020) Andromeda engine, incorporated into MaxQuant software, was used. The variable modifications selected as fixed modification in the processing were Acetyl (Protein N-Term), Oxidation (M), Deamidation (NQ), on the contrary the Carbamidomethyl (C).

### Enzyme-linked immunosorbent assay

To validate mass spectrometry results on microvesicle and exosome fractions we measured in the validation cohort the protein levels of Deoxyribonuclease 2 alpha (DNASE2) and Bisphosphate 3’-nucleotidase 1 (BPNT1) by commercially available ELISA kit for (MyBioSource) following the manufacturer’s instructions, whereas homemade ELISA was used to measure the SLK protein level. Briefly, microvesicles or exosomes were immobilized to the wells of nunc-immuno maxisorp plates (Thermo Fisher Scientific) at a concentration of 5 μg/well overnight at 4°C. Non-specific binding sites were blocked with 3% w/v BSA in PBS. After blocking, each well was washed three times with PBS-T (PBS with 0.05% v/v Tween-20) and incubated overnight at 4°C with anti-SLK monoclonal antibody (LifeSpan BioSciences). Plates were then washed and incubated with HRP-conjugated Mouse anti-Human IgG for 1 h. After extensive washes, the plates were incubated with the peroxidase substrate (TMB, Bio-Rad) and the absorbance was measured at 450 nm using a microplate reader (Bio-Rad).

### Statistical analysis

After normalization and missing value imputation with normal distribution, data obtained from mass spectrometry were analyzed by unsupervised hierarchical clustering using multidimensional scaling (MDS) with k-means and Spearman’s correlation to select outliers and the difference between samples. To identify the proteins that maximize the discrimination between BKV and Control in microvesicle or exosome, we applied *t*-test, machine learning methods such as non-linear SVM learning, and PLS-DA. For the *t*-test, the proteins that differed between the two groups with power of 80% and an adjusted *P*-value ≤ 0.05 after correction for multiple interactions (Benjamini-Hochberg) and a fold change ≥ 2 were considered to be statistically significant. In addition, an identity of 50% or more of the proteins and an area under the curve (AUC) in the received operating characteristic (ROC) analysis > 0.7 was considered acceptable.

Volcano plot displayed the statistical differences and the cutoff lines were established using the function y = c/(x-x0). In SVM learning, a fourfold cross-validation approach was applied to estimate the prediction and classification accuracy. Besides, SVM and PLS-DA generate, respectively, a rank and VIP-score (variable importance in projection) to establish a priority list of proteins to distinguish control and BKV samples in microvesicle and exosome fractions. Finally, gene set enrichment analysis (GSEA) was used to build a functional proteins network based on their Gene Ontology (GO) annotations using all identified proteins and those selected by the combined use of *t*-test, SVM and PLS-DA analysis. The protein profile expression data were loaded in the dataset and a ranked list was assigned to each GO annotation term. These ranks take into account the number of proteins associated with each GO annotation term with respect to all proteins, their mean of fold change and the *P*-value after FDR correction for multiple interactions. These ranks are confined between –1 and 1, corresponding to minimal and maximal enrichment in each group.

For the ELISA assay, Kruskal-Wallis test for unpaired samples was used to assess the difference in the concentration of the potential biomarkers. Results were expressed as medians and interquartile range (IQr). ROC curves were generated to assess the diagnostic efficiency of each assay. AUC value was classified as: 0.5, not discriminant; 0.5–0.6, fail; 0.6–0.7, poor; 0.7–0.8, fair; 0.8–0.9, good and 0.9–1, excellent. Youden’s index was used to identify the cutoff of each assay. For Kruskal-Wallis test data was considered to be statistically different with two sides *p*-values ≤ 0.05 after Dunn’s correction for multiple comparison.

All statistical tests were carried out using Origin Lab V9 and R.

## Results

### Characterization of exosomes and microvesicles

Dynamic Light Scattering was used to determine purity and size of the EVs. Gaussian distribution profile with peak means at 1,000 ± 65 and 90 ± 5 nm revealed the typical size for microvesicles and exosomes, respectively (Supplementary Figures 1A,B).

### Protein composition of extracellular vesicles of kidney transplant recipients with BK virus infection and controls without infection

The protein content of urinary microvesicles and exosomes from patients with BKV infection and CTR was assessed by mass spectrometry.

**FIGURE 1:**
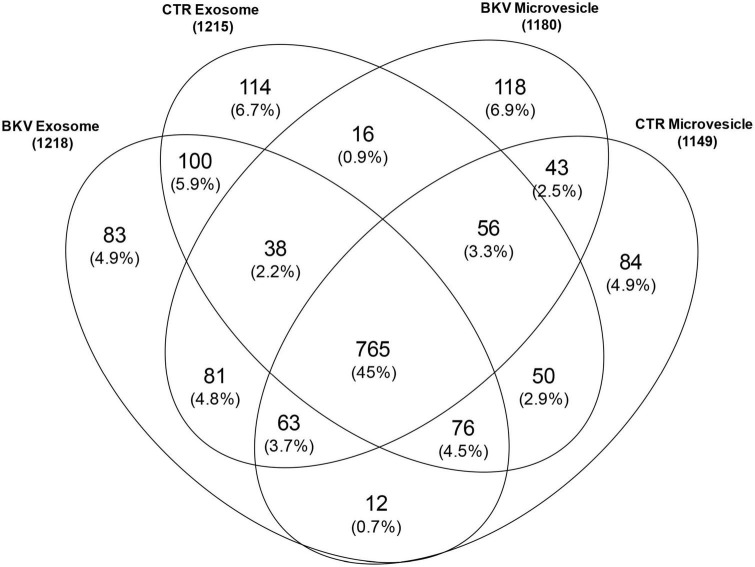
Venn diagram of total proteins identified in urinary microvesicles and exosomes of patients with BKV infection and patients with no infection (CTR). The diagram shows common and exclusive proteins in 12 patients with BKV infection and 6 CTR by mass spectrometry. The numbers (and percentages) of proteins in the overlapping and non-overlapping areas are indicated. Overall, 118 and 83 proteins were unique for microvesicles and exosomes in patients with BKV infection. In the CTR group 84 and 114 proteins were unique for the microvesicles and exosomes, respectively.

We identified 1699 proteins in total (Supplementary Table 1) and 765 (45%) were common to all four sample types. In the urinary EVs isolated from patients with BKV infection, we identified 118 (6.9%) and 83 (4.9%) unique proteins for microvesicles and exosomes, respectively. In the CTR group 84 (4.9%) and 114 (6.7%) proteins were unique for the microvesicles and exosomes, respectively (Figure 1).

The proteins in the microvesicles from CTR and the group of patients with BKV infection derived from several cell components: 49% derived from membranes, 27% from the cytoplasm, and 24% from the nucleus. Likewise in the exosomes: 46% of proteins derived from membranes, 28% from the cytoplasm, and 26% from the nucleus.

### Functional analysis of total proteins in urinary extracellular vesicles of kidney transplant recipients with BK virus infection and controls without infection

**FIGURE 2:**
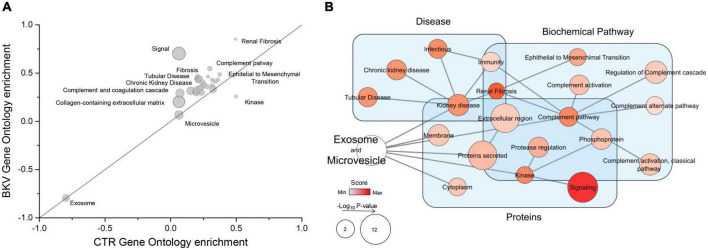
Gene Ontology (GO) enrichment analysis and Gene Set Enrichment Analysis (GSEA) of the total proteins identified. **(A)** Plot shows the GO enrichment of total proteins in exosomes and microvesicles isolated from urine of patients with BKV infection and CTR. In the graph, the points located on the straight line passing through the coordinates (1x, 1y) and (-1x, -1y) are the equally enriched signatures, while those above or under this line are positively enriched in BKV or CTR, respectively, (see detail in Supplementary Table 2). The distance from this line is proportional to the enrichment score in one of the two groups. The size of circles is proportional to the probability after correction for multiple comparison. **(B)** Network of GSEA results. GSEA is a computational method that determines whether an *a priori* defined set of proteins shows statistically significant differences between two phenotypes. In particular, the diagram summarizes the GSEA results mapped as a network. Nodes and edges represent, respectively, the GO annotation terms and their interaction. The color intensity of each node indicates the level of enrichment in BKV samples and size is proportional to its *p*-value. “Signal” is the annotation term with the highest enrichment score. In addition, the different GO terms were clustered in the function of their GO annotation (light blue rectangles).

To determine the biological functions of the proteins identified by mass spectrometry, we performed a GSEA. This analysis demonstrated that 1, 27 and 2 biological processes were, respectively, enriched in CTR, patients with BKV infection or both conditions (Figures 2A,B and Supplementary Table 2). The biological processes enriched in patients with BKV infection included immunity, complement activation, renal fibrosis, tubular diseases, epithelial to mesenchymal transition, whereas the only GO annotation term including proteins less abundant in patients with BKV infection compared to CTR was “kinase” (Figures 2A,B and Supplementary Table 2).

**FIGURE 3:**
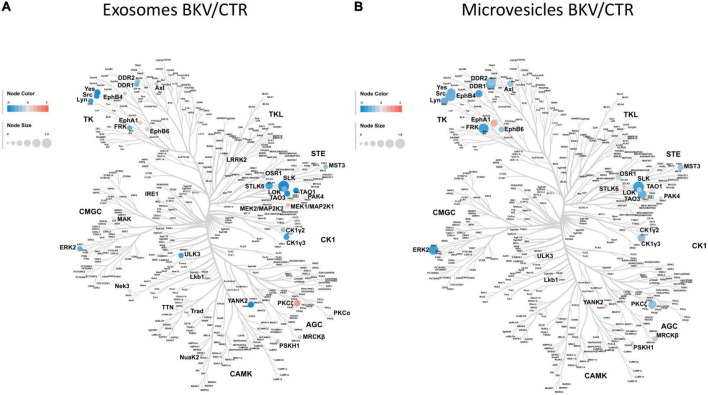
Kinome profile of microvesicles and exosomes isolated from urine of patients with BKV infection and Control. In these diagrams, featuring a dendrogram of human protein kinases, the log_2_ of label-free quantification intensity fold change of each kinase (circle) was converted by a pseudocolor scale with red, blue, and white indicating, respectively, up, down and non-differentially expressed proteins in **(A)** exosomes and **(B)** microvesicles isolated from 12 patients with BKV infection and 6 Control. The size of circles was proportional to the corresponding –Log_10_ *p*-value. Kinome profiles showed a significant down-regulation of kinases in patients with BKV infection compared to CTR in both extracellular vesicles. SLK was able to reach the higher level of discrimination between the 2 study groups in both extracellular vesicles.

The kinome analysis by CORAL tree application (20) demonstrated that a large number of kinases were down-regulated in both exosomes (Figure 3A) and microvesicles of patients with BKV infection (Figure 3B) compared to CTR. STE20-Like Kinase (SLK) was able to reach the higher level of discrimination between the 2 study groups.

### Proteomic profile discriminates patients with BK virus infection from controls without infection

**FIGURE 4:**
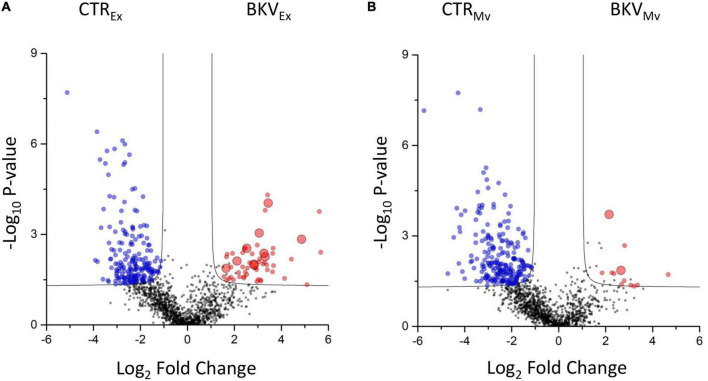
Volcano plot showing the univariate statistical analysis of exosomes and microvesicles from patients with BKV infection and control (CTR). The plots are based on the fold change (log_2_) and the *P*-value (–log_10_) of all proteins identified in **(A)** exosomes and **(B)** microvesicles. Red, blue and black circles represent up-regulated, down-regulated and non-differentially expressed proteins, respectively, in patients with BKV infection vs. CTR. Black line indicates the limits of statistical significance (adjusted *p*-value ≤ 0.05 and fold change ≥ 2).

*T*-test was used to identify top discriminative proteins between KTRs with BKV infection and CTR. Statistical analysis revealed a total of 427 discriminatory proteins, 251 that distinguished BKV from CTR in exosomes (Figure 4A and Supplementary Table 3) and 239 in microvesicles (Figure 4B and Supplementary Table 3).

**FIGURE 5:**
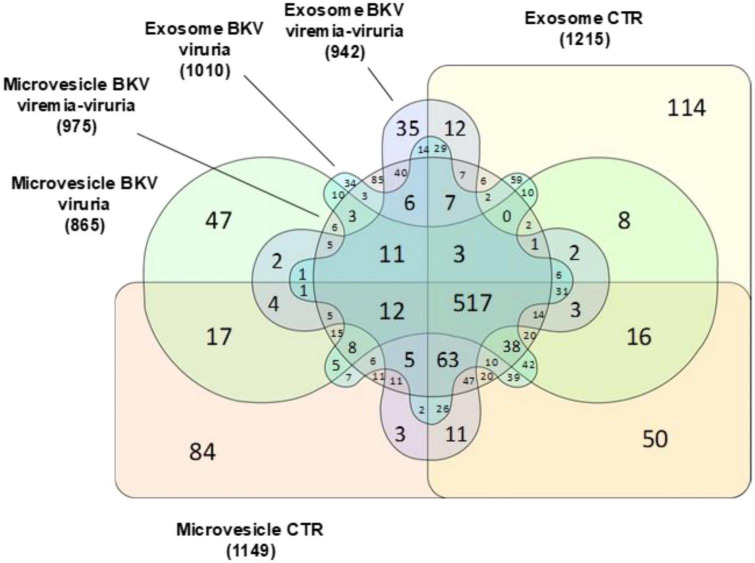
Venn diagram of total proteins identified in microvesicles and exosomes stratified in patients with BKV viremia-viruria, BKV viruria and control (CTR). Venn diagram shows the distribution of the total proteins identified as well as the common and exclusive proteins in urinary microvesicles and exosomes isolated from patients with BKV viremia-viruria, BKV viruria and CTR. The numbers represent the distinct proteins in the overlapping and non-overlapping areas. Among the proteins identified, 47, 34, 85, and 35 were exclusively found in microvesicles of KTRs with BKV viruria, exosomes of KTRs with BKV viruria, microvesicles of KTRs with BKV viremia-viruria and exosomes of KTRs with BKV viremia-viruria, respectively.

Notably, among the proteins identified in urinary EVs isolated from KTRs with BKV infection, 47 (2.8%), 34 (2%), 85 (8.7%), and 35 (2.1%) proteins were exclusively found in microvesicles of KTRs with BKV viruria, exosomes of KTRs with BKV viruria, microvesicles of KTRs with BKV viremia-viruria and exosomes of KTRs with BKV viremia-viruria, respectively (Figure 5).

**FIGURE 6:**
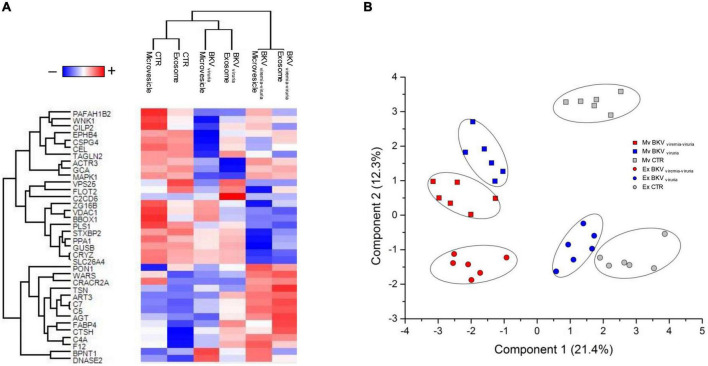
Heatmap and partial least squares discriminant analysis (PLS-DA) of proteins that maximize the discrimination among all condition. **(A)** Heatmap, which depicts values for a main variable of interest across two axis variables as a grid of colored square, of 36 proteins identified through the combined use of univariate statistical analysis, machine learning and PLS-DA. In the heatmap, each row represents a protein, and each column corresponds to a condition. Normalized Z-scores of protein abundance are depicted by a pseudocolor scale with red representing up-regulation and blue indicating down-regulation. The dendrogram displays the outcome of unsupervised hierarchical clustering analysis, placing similar proteome profile values near each other. Visual inspection of the dendrogram and heatmap demonstrates the ability of these proteins to distinguish among the different conditions. **(B)** Two-dimensional scatter plot of PLS-DA of proteins maximizing the discrimination among all condition. Plot shows microvesicles (squares) and exosomes (circles) of CTR (gray), BKV viremia-viruria (red), and BKV viruria (blue) using the proteins identified by the combined use of *t*-test, SVM and PLS-DA. Ellipsis indicates 95% confidence interval. Visual inspection of scatter plot shows the ability of these proteins to clearly distinguish among the different conditions.

SVM and PLS-DA were applied to generate a priority list of all statistically significant proteins to distinguish KTRs with BKV infection from CTR in the microvesicles and exosomes (Supplementary Table 1). The profile of proteins that maximize the discrimination between patients with BKV infection and CTR, and/or patients with BKV viremia-viruria and BKV viruria alone for microvesicles and exosomes, after Z-score normalization, were reported in Figures 6A,B.

The proteins that achieved maximum discrimination between KTRs with BKV and CTR (in both EVs) were DNASE2, F12, AGT, CTSH, C4A, C7, FABP4 and BPNT1 (Figure 6A).

Nevertheless, although a set of proteins were able to differentiate patients with BKV viruria from those with BKV viremia-viruria, the urinary proteomic profile of these patients resulted quite similar.

### Validation of proteomics analysis by enzyme-linked immunosorbent assay

To confirm the mass spectrometry results, we measured the urinary protein level of SLK, DNASE2 and BPNT1 (top discriminating proteins) by ELISA in microvesicles and exosomes isolated from patients with BKV infection and CTR included in the validation cohort.

**FIGURE 7:**
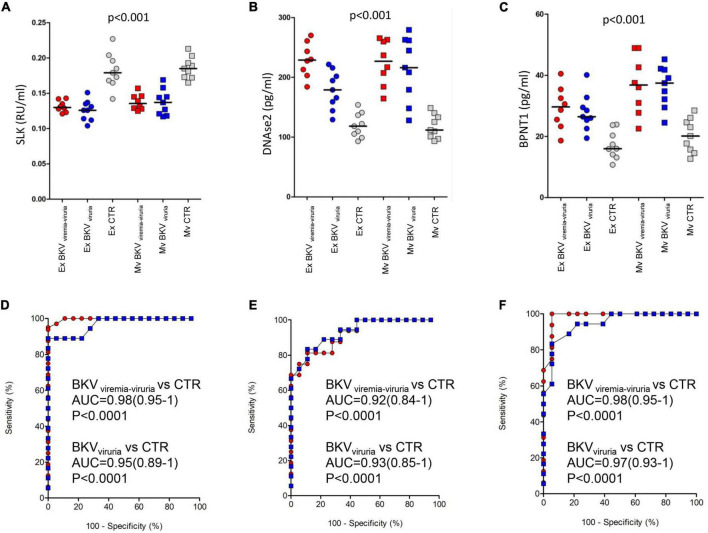
ELISA of STE20 Like Kinase (SLK), Deoxyribonuclease 2 (DNASE2), and 3’(2’)5’-bisphosphate nucleotidase 1 (BPNT1). Plots show the median (black line) obtained from each duplicate measurement of **(A)** SLK, **(B)** DNASE2, and **(C)** BPNT1 in exosomes (circle) and microvesicles (square) isolated from urine of control (gray) and patients with BK viruria alone (blue) and BK viruria and viremia (red). Patients with BKV infection have a significant lower level of SLK compared to CTR in both extracellular vesicles. The opposite trend is observed for DNASE2 and BPNT1. ROC curve analysis for **(D)** SLK, **(E)** DNASE2, and **(F)** BPNT1 ELISA comparing control and patients with BK viruria alone (blue square) and BK viruria and viremia (red circle).

As showed in Figure 7A, we found a significantly lower level of SLK in both exosomes and microvesicles of patients with BKV infection compared to CTR (*p* < 0.001), whereas the opposite trend was observed for DNASE2 and BPNT1 (Figures 7B,C) (*p* < 0.001). Also in this case, no difference was found between patients with BKV viruria vs. patients with BKV viruria and viremia.

ROC curves demonstrated the capability of the 3 proteins to clearly discriminate KTRs with BKV infection vs. CTR and their potential employment as new disease biomarkers (whether validated in a larger patients’ cohort) (Figures 7D–F).

## Discussion

The BKV infection is a complex and multi-factorial pathological condition following kidney transplantation that represents one of the leading causes of kidney allograft loss. The reported incidence of this infection varies from 10 to 40% (4–7) with irreversible graft failure in 10–80% of cases in presence of BKVAN (21). There is a considerable variability in the incidence and outcome of BKVAN which may reflect differences in diagnostic criteria, the immunosuppressive protocol and management strategies across renal transplant centers.

Several biological pathways (including Akt-mTOR-S6 kinase axis) may be activated in the kidney during this viral infection and are necessary to ensure the virus replication/dissemination (22) and they may activate a virus-related immune-inflammatory network leading to the release of cytokines and chemokines able to induce an accelerated chronic allograft damage.

Histopathological studies have demonstrated that in KTRs, BKV infection may induce viral cytopathic changes in the kidney tubular epithelia with consequent tubular cell necrosis, and cellular inflammation, tubular atrophy and fibrosis (23). However, most of the described features are detected in a late stage of the disease (when BKVAN is histologically diagnosed). Contrarily, the pathophysiological machinery activated in the early phase of BKV infection is only partially defined.

To address this knowledge gap and to identify new biological factors involved in the BKV infection (also in the early stage), we employed an innovative high-throughput technology to compare the whole protein content of urinary EVs (microvesicles/exosomes) isolated from KTRs with BKV infection with normal renal function from those obtained by KTRs without infection or other clinical allograft disorders (controls).

GSEA revealed that a large number of biological processes were enriched in the urinary EVs isolated from KTRs with BKV infection compared to controls including renal fibrosis, epithelial to mesenchymal transition (EMT), immunity and complement activation.

The activation of EMT (a process during which renal epithelial tubular cells lose cell-cell juncion and cell polarity and acquire mesenchymal phenotypes with migratory and invasive properties), and fibrosis, during the BKV infection was expected, since has been previously reported the upregulation of several genes associated with EMT in kidneys of patients with BKVAN (24). Since our results have been obtained in patients with BKV infection and normal allograft function, it is conceivable that the virus could trigger these events in the early phase of the infection. However, further studies are necessary to address this issue.

Also, the involvement of immunity in this viral disease has been previously reported. The innate response by means of natural killer, gamma-delta T cells and neutrophils has an important role in BKV infection (25). The double-stranded DNA (dsDNA) of BKV is recognized by intracellular sensors such as retinoic acid-inducible gene I (RIG-I)-like receptors, Toll-like receptor 3 (TLR3), melanoma differentiation-associated gene 5 (MDA5) and nucleotide oligomerization domain-like receptors enhancing the expression of cytokines, chemokines and type I interferons (IFNs) (26–29) to control infection. This could also result in tissue injury and dysfunction contributing to BKVAN (30).

The role of humoral immunity in the regulation of BKV activity is controversial. Specific antibodies against BKV have been found in 82% of healthy subjects (31). In KTRs with BKV viruria or viremia, there is a correlation between BKV-specific antibody responses and severity of infection (32–34) compared to patients without BKV infection. Studies evaluating the correlation between BKV antibody level pre-transplant and intensity of infections have shown that the mean BKV-antibody level before transplantation was lower in KTRs who developed viremia compared to patients who never developed viremia (35–37). However, even seropositive recipients developed sustained viremia suggesting that humoral response cannot protect from reactivation of viral replication after transplantation (33, 38). Meanwhile, KTRs received the graft from seropositive donors have a higher level of BKV-specific-antibodies after transplantation compared to recipients from seronegative donors (39) indicating a possible ability of BKV infection from the donor to induce the humoral immune response.

Instead, data on the role of complement system in BKV infection is still lacking. The up-regulation of complement factors (such as C4d) in patients with BKV infection has been only supposed by the increased number of complement-related rejection after BKVAN (40). As in other kidney disorders, complement may regulate the immune response and, if significantly activated, induce the onset of acute cellular rejection and accelerate the microvascular damage.

Our bioinformatics analysis, revealed that a large number of kinases were down-regulated in EVs of patients with BKV infection compared to CTR revealing a potential defense mechanism by which allograft cells may try to antagonize virus proliferation and to enhance tissue-anti-viral response (41).

Interestingly, among the identified kinases, STE20 Like Kinase (SLK), a protein involved in cell polarization (42) cytoplasmic microtubule organization (organization of cytoplasmic microtubules) (43), cell cycle progression (44), and apoptosis (45) resulted the most significantly down-regulated in patients with BKV infection compared to CTR. Although there are no previous data, it is plausible that low content of SLK play a role in BKV infection by inducing a G2/M-phase arrest in infected renal tubular epithelial cells (44, 46, 47) and probably by affecting the microtubule organization necessary for the virus movement (43, 48).

Interestingly, then, statistical analysis demonstrated that two proteins resulted significantly up-regulated in urinary EVs of KTR with BKV infection (both viruria alone and BK viruria and viremia) compared to CTR: Bisphosphate 3′-nucleotidase 1 (BPNT1) and Deoxyribonuclease 2 alpha (DNASE2).

BPNT1 is involved in the sulfation process wherein the universal sulfate donor phosphoadenosine-phosphosulfate (PAPS) transfers, by sulfotransferase enzymes, a sulfate group to a target substrate. BPNT1 converts 3′-phosphoadenosine 5′-phosphate (byproduct of the reaction) to 5’-AMP (49). Although poorly described in kidney disorders, this enzyme has been associated with alteration in uromodulin levels (50). Concentration of uromodulin (also known as Tamm–Horsfall protein) is correlated to the formation, in the kidneys, of polyomavirus-haufen. The high concentration of uromodulin determines the aggregation of polyomavirus that can be detected and serve as specific biomarker for BKVAN with positive and negative predictive values of 97 and 100%, respectively, (51, 52).

In addition, the up-regulation of DNASE2, an enzyme which digests in lysosomes DNA of phagocytosed apoptotic bodies or DNA entering the cell via endocytosis (53, 54) could target the viral DNA for degradation (55).

In conclusion, the present pilot study, although performed in a small number of patients, provided an integrated overview of the pathophysiological fingerprints associated with the BKV infection in kidney transplantation, and it demonstrated the activation of virus-related biological mechanisms in the allograft even in patients with BKV viruria alone and without allograft functional impairment. Therefore, a preventive therapy (mainly reduction of the immunosuppressive therapy) should be considered also in patients with BK viruria alone. Additional proteomic analysis including patients with different stages of infection and with BKVAN should be performed in future. Moreover, some of our identified proteins (including SLK, BPNT1, and DNASE2), whether validated in larger cohorts of patients, could turn to be potential new early disease biomarkers and novel therapeutic targets.

## Data availability statement

The mass spectrometry data were deposited to the ProteomeXchange Consortium (http://www.proteomexchange.org/) via the PRIDE partner repository under the data set identifier: PXD035494.

## Ethics statement

This study was reviewed and approved by the Ethics Committee of Verona and Rovigo provinces, Italy. The patients provided their written informed consent to participate in this study.

## Author contributions

MB, SG, and GZ concepted and designed the study, analyzed the data, and wrote the manuscript. MB, SG, and GZ analyzed the data and wrote the manuscript. GG and GS contributed to manuscript revision. All authors contributed to the article and approved the submitted version.
