# Supplementary material for: Proteomic analysis of urinary extracellular vesicles of kidney transplant recipients with BKV viruria and viremia: A pilot study
Source: Front Med (Lausanne). 2022 Nov 17;9:1028085. doi: 10.3389/fmed.2022.1028085 (PMC9712214; doi:10.3389/fmed.2022.1028085)
Supplement: Supplementary file 1 [file Data_Sheet_1.PDF]

Supplementary Material

Supplementary Results

Characterization of exosomes and microvesicles

Purity and size of the EV isolated by ultracentrifugation were confirmed by DLS, revealing a Gaussian distribution profile with peak means at 1000±65 and 90±5 nm, the typical size for microvesicles and exosomes, respectively (Supplemental Figure 1A, B).

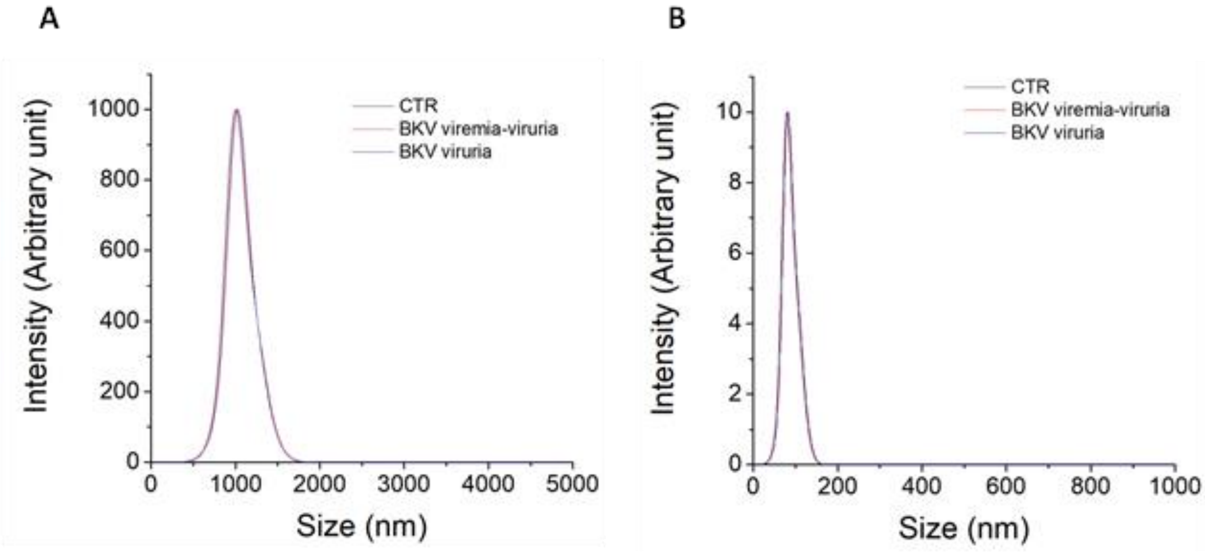

Supplementary Figure 1. Size characterization of microvesicle and exosomes purified from urine of control and patients with BK infection. Plots show the size distribution of **(A)** microvesicles and **(B)** exosomes, as evaluated by dynamic light scattering. The plots show a Gaussian distribution profile with a mean peak at 1000±65 and 90±5 nm for microvesicles and exosomes respectively. No statistical differences were observed between the exosomes or microvesicles isolated from control and patients with BK infection.

.
